# Snail shell colour evolution in urban heat islands detected via citizen science

**DOI:** 10.1038/s42003-019-0511-6

**Published:** 2019-07-19

**Authors:** Niels A. G. Kerstes, Thijmen Breeschoten, Vincent J. Kalkman, Menno Schilthuizen

**Affiliations:** 1 Naturalis Biodiversity Center, 2333CR Leiden, The Netherlands; 2 EIS Kenniscentrum Insecten, 2333CR Leiden, The Netherlands; 3 Institute for Biology Leiden, Leiden University, 2333BE Leiden, The Netherlands; 4 Present Address: Biosystematics Group, Wageningen University & Research, Droevendaalsesteeg 1, 6708 PB Wageningen, The Netherlands

**Keywords:** Evolutionary genetics, Urban ecology

## Abstract

The extreme environmental conditions that prevail in cities are known to cause selection pressures leading to adaptive changes in wild, city-dwelling, organisms (urban evolution). The urban heat island, elevated temperatures in the city centre due to a combination of generation, reflection, and trapping of heat, is one of the best recognised and most widespread urban environmental factors. Here, we use a citizen-science approach to study the effects of urban heat on genetically-determined shell colour in the land snail *Cepaea nemoralis* in the Netherlands. We use smartphone applications to obtain colour data on almost 8000 snails throughout the country. Our analysis shows that snails in urban centres are more likely to be yellow than pink, an effect predicted on the basis of thermal selection. Urban yellow snails are also more likely to carry dark bands at the underside of the shell; these bands might affect thermoregulation in yet underexplored ways.

## Abstract (web summary)

Niels Kerstes et al. report a citizen science effort to record the shell colour and banding patterns of nearly 8000 *Cepaea nemoralis* snails in the Netherlands to understand the potential evolutionary effect of urban environments on that snail species. They find that snails in urban centres are more likely to be yellow than pink, which may be explained by thermal selection.

## Introduction

It is increasingly understood that the environmental changes that humans are causing in a growing part of the earth's surface, especially in heavily urbanised areas, are becoming a major evolutionary force for the organisms inhabiting those places^1,2^. Numerous examples exist of such urban evolution, e.g., species of city-dwelling animals and plants adapting to urban microclimate, urban habitat fragmentation, and urban pollution^3,4^.

Urban evolution is a process that, by definition, takes place in areas densely populated by people. Moreover, since certain aspects of the urban environment (such as the urban heat island^5^) are shared by cities worldwide, different cities could be viewed as replicates for studying specific evolutionary responses in widespread species^6^. For these reasons, urban evolution is eminently suited to be studied through the dispersed monitoring systems that citizen science provides (where citizen science refers to the gathering of scientifically relevant data by the general public). However, although the 2009 Evolution Megalab (www.evolutionmegalab.org^7^) has shown that citizen science methods enable the tracking of non-urban contemporary evolutionary change, urban evolution has not yet been targeted by any major citizen science projects.

To make a citizen science approach to urban evolution possible, several conditions need to be met^8,9^. First of all, the organism of study must be widespread, harmless, common, large enough to be observed and studied without the need of specialised equipment, and carry externally visible genetic variation that responds to a universal urban selection pressure. Second, a simple digital data collection platform needs to be present to enable a large number of untrained citizen scientists to upload data. Finally, some pre-existing knowledge on the evolutionary ecology of the species in question is required for hypotheses to be formulated and tested.

In this paper, we describe such a citizen science approach to urban evolution in the Netherlands. We developed a simple smartphone app (SnailSnap) for taking and uploading images of the shell colour polymorphism in *Cepaea nemoralis*, an intensively-studied land snail that has been a model species in evolutionary ecology and genetics for over a hundred years^10,11^. The app was linked to a popular Dutch citizen science platform, which allowed us to test the hypothesis that the urban heat island results in an evolutionary response in genetically determined snail shell colour in *Cepaea nemoralis*. Such a response would be expected since shell colour (which ranges from pale yellow to dark brown, and may involve up to 5 black spiral bands) is known to be related to the ability of the snail to withstand extreme temperatures. Our analysis of colour data on almost 8,000 snails photographed throughout the Netherlands shows that snails in urban centres are more likely to be yellow than pink. Urban yellow snails are also more likely to carry dark bands at the underside of the shell; these bands might affect thermoregulation in yet underexplored ways.

## Results

### Snailsnap

**Fig. 1:**
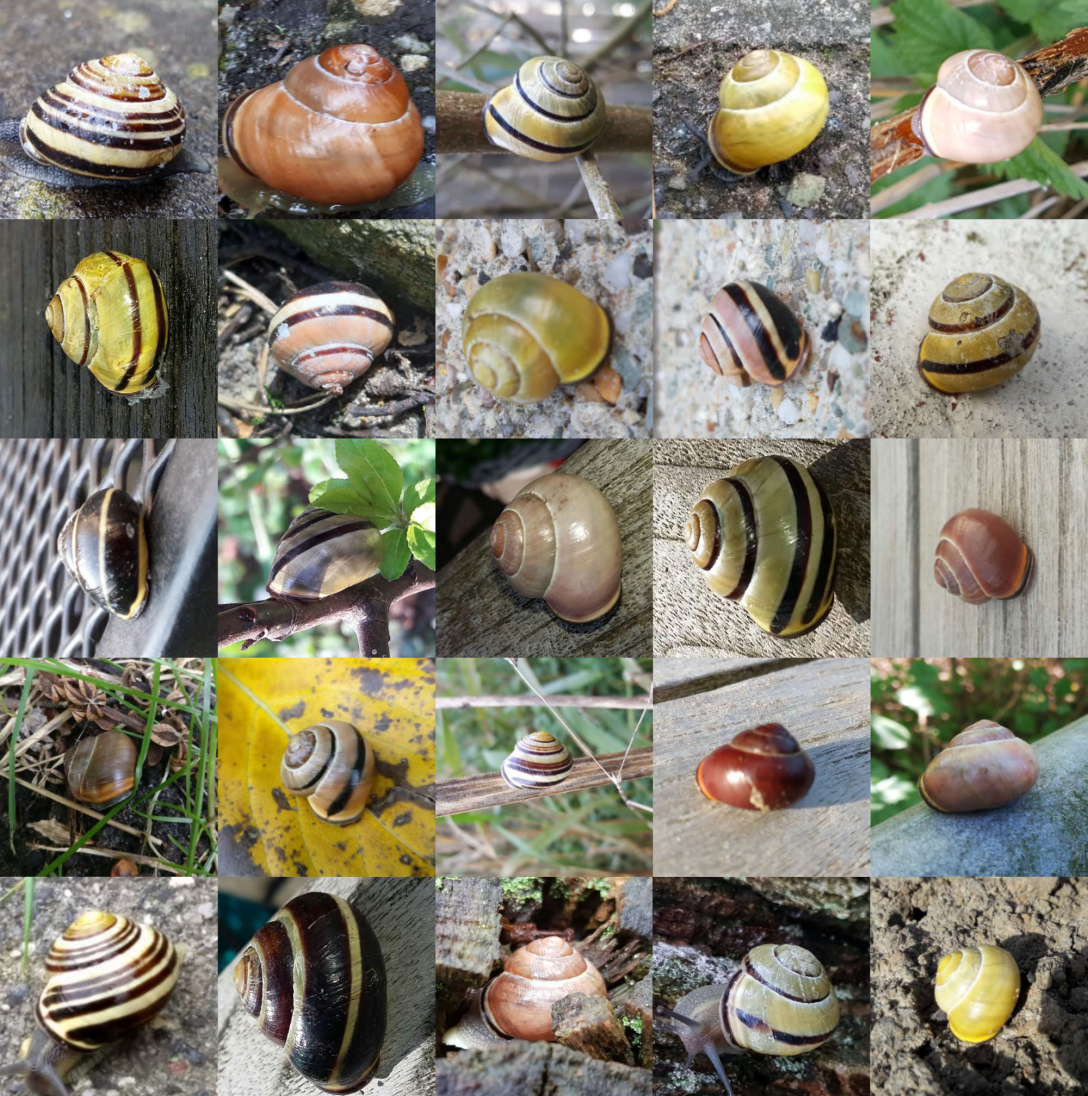
Examples of photos of *Cepaea nemoralis* as submitted by citizen scientists

The SnailSnap app was downloaded 1180 times, and 9483 images of *C. nemoralis* were uploaded (see Fig. 1 for sample images). After removal of pictures that could not be unequivocally categorized (see “Methods” section), we retained 7868 images of unique, individual snails and investigated the relationship between genetically-based shell colour and four habitat types: agricultural land; nature, including forests; urban green areas, i.e., urban parks and forests, sport and recreational areas; and urban “grey” areas, i.e., residential, commercial, and industrial areas.

### Accuracy of the Snailsnap data

**Fig. 2:**
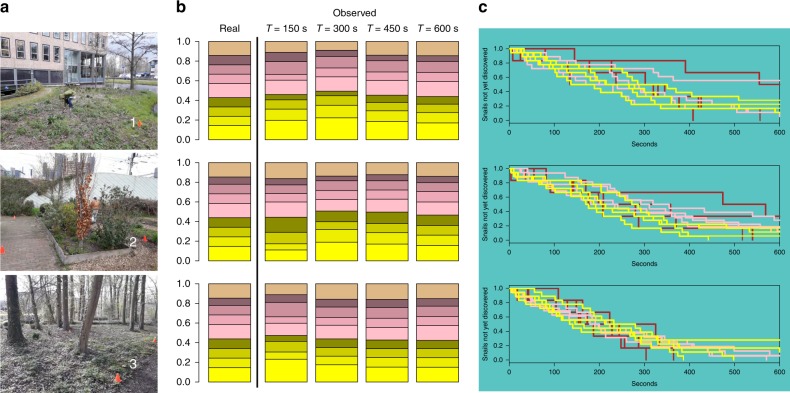
Pictures showing the three locations (**a**), stacked bar plots showing the real proportions of the distributed shells and the proportions found after four increasing time periods (average of the four volunteers, **b**), and Kaplan–Meier curves showing what proportion of shells was not yet discovered (**c**). In **c**, each line represents shells of a certain colour (yellow, pink or brown) per volunteer (per location and per colour, four lines are shown)

**Fig. 3:**
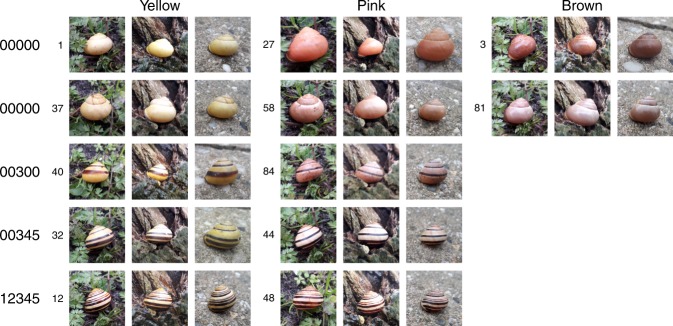
Examples of pictures of the same shells (the smaller numbers indicate shell-id) taken against the three different backgrounds (soil/vegetation, tree, concrete)

Results for experiment 1 are presented in Fig. 2. After ten minutes, the proportions of shells found match the ‘real’ proportions. Before that, proportions fluctuate, but there appears to be no strong bias towards a certain colour. Even after only 150 s, proportions of colours (irrespective of banding) approach the real proportions. In experiment 2, the classification of the actual shell specimens by M.S. matched the classification of shells on the pictures by 94% (soil/vegetation), 93% (tree) and 94% (concrete). It should be noted that there was no 100% agreement between the classification of shell specimens by M.S. and N.A.G.K. (viz., 98%). Exact Multinomial tests were used to compare the classifications (yellow, pink or brown) of actual shell specimens by M.S. with the classification based on pictures (actual shells vs. ‘tree’ photos, actual shells vs. ‘concrete’ photos and actual shells vs. ‘soil/vegetation’ photos). No significant differences were found for actual shells vs. ‘tree’ photos (p (distance measure) = 0.0039, *p* = 0.7531), actual shells vs ‘concrete’ photos (p (distance measure)= 0.0026, *p* = 0.213) and actual shells vs. “soil/vegetation” photos (p(distance measure) = 0.0026, *p* = 0.213), and. Against the tree-background, one brown and two pink snails were classified as yellow, two brown were classified as pink, and one pink was classified as brown. Figure 3 shows examples of pictures of the same shells photographed against the three different backgrounds.

### Colour

**Fig. 4:**
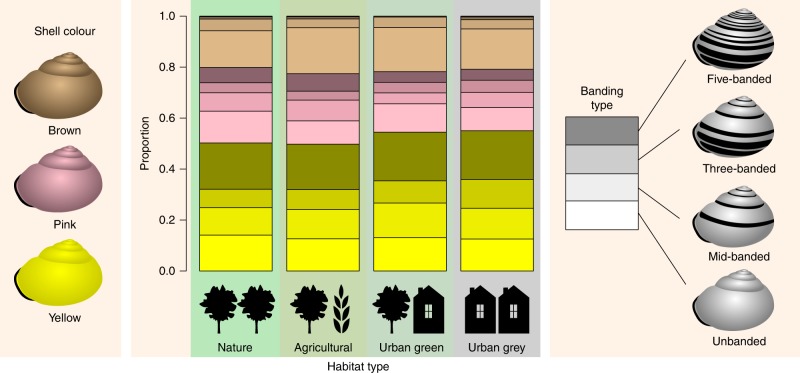
Proportions of shell colour (yellow, pink or brown) and the four main banding types per habitat type

We found that the proportion of yellow snails is higher in urban than in non-urban areas (Fig. 4, χ^2^ = 12.35, df = 1, *p* = 0.0004), while the proportion of pink snails is lower (χ^2^ = 14.21, df = 1, *p* = 0.0002). The proportions of brown snails did not differ significantly (χ^2^ = 1.96, df = 3, *p* = 0.58). There was no significant difference in the proportion of yellow snails between urban grey and urban green areas (χ^2^ = 0.08, df = 1, *p* = 0.77). The proportion of yellow three-banded snails in the urban grey habitat was higher than in agricultural (χ^2^ = 8.78, df = 1, *p* = 0.003), natural (χ^2^ = 12.91, df = 1, *p* = 0.0003), or urban green areas (χ^2^ = 4.78, df = 1, *p* = 0.029).

**Fig. 5:**
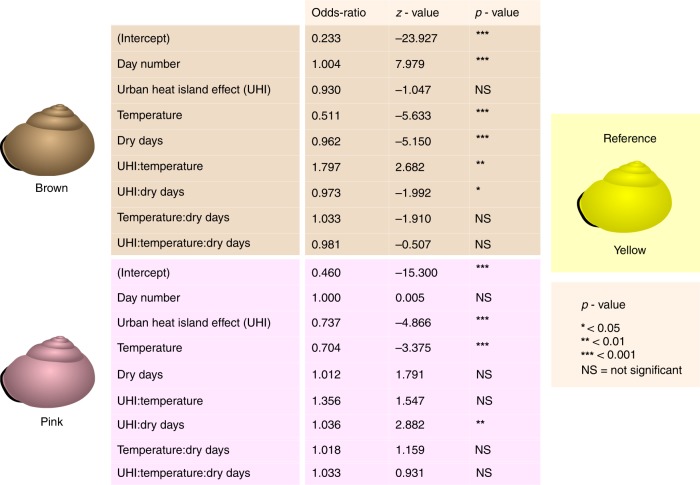
Results of a multinomial regression model with colour as outcome variable. The includes snails from all land use types (*n* = 7,868). Yellow snails are the reference

The results of the multinomial logistic regression with shell colour as outcome variable (Fig. 5) provide further explanation for these patterns. Yellow was chosen as the reference colour. When including snails from all habitat types, the probability of a pink or a brown snail decreases with increasing temperature relative to the probability of a yellow snail (Odds-ratios < 1).

**Fig. 6:**
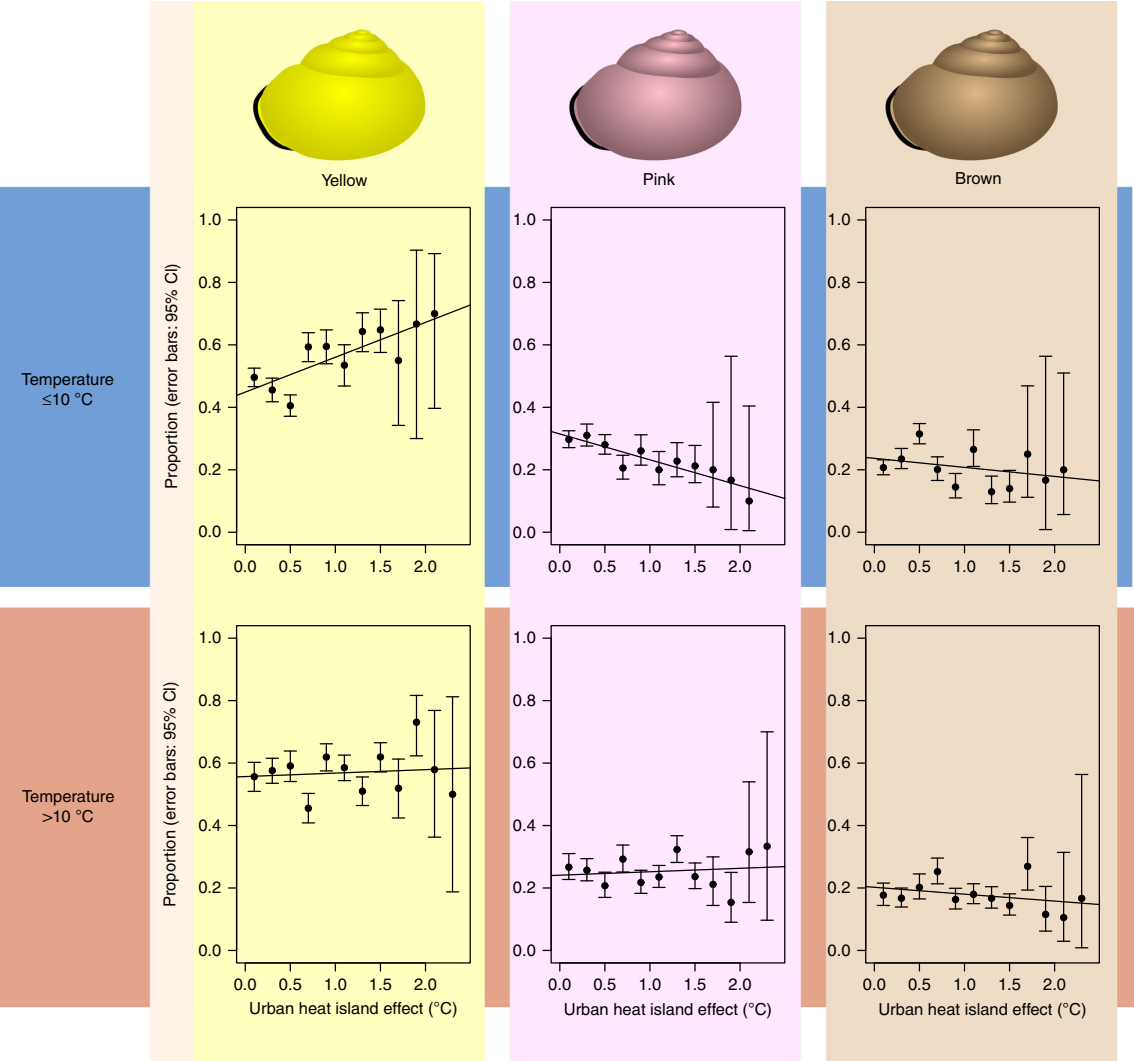
The proportions of yellow, pink and brown snails as a function of the Urban Heat Island effect (UHI). Error bars indicate 95% confidence intervals (Wilson score interval). Based on the median average temperature (10 °C), the data set was split into a “cold” half (A, *n* = 3936) and a “hot” half (B, *n* = 3932). Starting at UHI = 0 °C, UHI was divided into categories of 0.2 °C and proportions per category were plotted against the central value of each category. Linear regression lines are included for illustrative purposes

UHI (Urban Heat Island effect) is a significant predictor for the probability of a pink versus a yellow snail. There is also a significant interaction between UHI and the number of dry days for the probability of a pink versus a yellow snail. At sites with relatively low numbers of dry days, the proportion of pink snails decreases with increasing UHI. At sites with a relatively high number of dry days, UHI does not result in a decrease in the probability of pink snails. For the probability of a brown snail, there is a significant and fairly strong interaction between UHI and temperature (Fig. 6). Furthermore, the probability of a brown snail decreases with an increasing number of dry days (Supplementary Fig. 1). When only snails from urban grey areas are included, we still find a relationship between UHI and shell colour (Table 1). The proportion of brown snails increases with increasing day number.

**Table 1:** Results of a multinomial regression model with colour as outcome variable, including only snails from urban grey areas (Urban grey, *n* = 4687)

		Urban grey		
		Odds-ratio	*z*-value	*p*-value
*Brown*	(Intercept)	0.219	−19.177	***
	Day number	1.004	5.904	***
	UHI	0.844	−1.693	NS
	Temperature	0.574	−3.549	***
	Dry days	0.962	−3.966	***
	UHI:Temperature	1.917	2.179	*
	UHI:Dry days	1.008	0.412	NS
	Temperature:Dry days	1.017	0.749	NS
	UHI:Temperature:Dry days	0.949	−0.972	NS
*Pink*	(Intercept)	0.395	−13.941	***
	Day number	1.001	1.241	NS
	UHI	0.719	−3.592	***
	Temperature	0.753	−2.091	*
	Dry days	1.029	3.357	***
	UHI:Temperature	1.758	2.079	*
	UHI:Dry days	1.039	2.171	*
	Temperature:Dry days	1.030	1.518	NS
	UHI:Temperature:Dry days	1.009	0.192	NS

Yellow snails are the reference

*P*-values: *< 0.05; **< 0.01; ***< 0.001; NS = not significant

### Banding

**Fig. 7:**
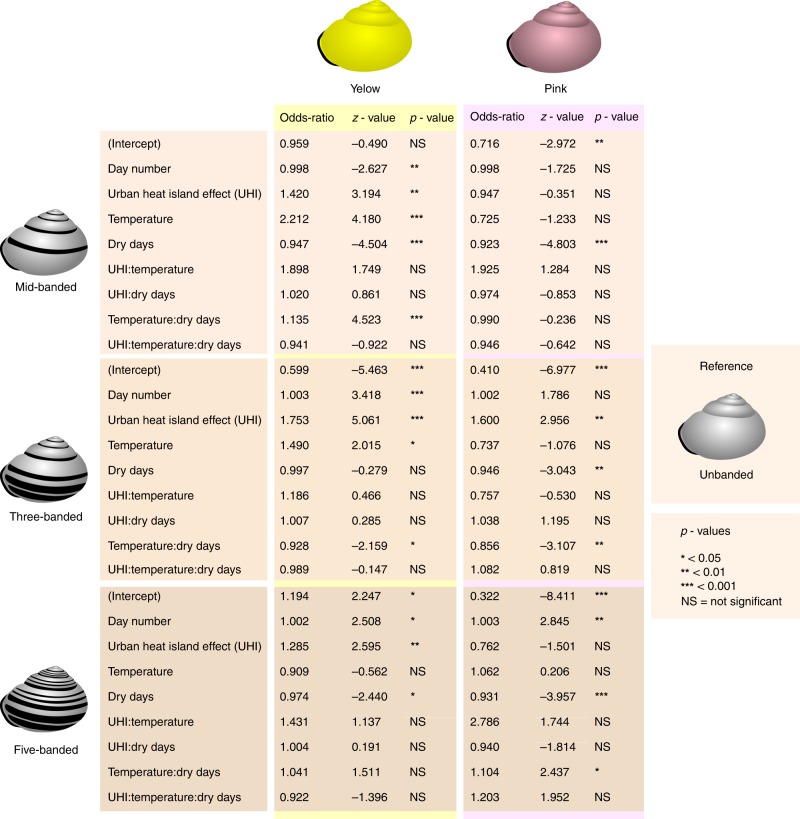
Results of multinomial regression models with banding type. Unbanded, mid-banded, three-banded or five-banded were used as outcome variable, for yellow (*n* = 3660) and pink (*n* = 1695) snails. Unbanded snails are the reference

Since brown snails are rarely banded, multinomial regression models with banding type as outcome variable were only performed for yellow and pink snails (Fig. 7). “Unbanded” was chosen as the baseline banding type. The probabilities of unbanded and five-banded yellow snails decrease with increasing temperature, due to increases in the probabilities of mid-banded (at sites with a relatively high number of dry days) and three-banded snails. The probability of an unbanded yellow snail decreases with increasing UHI. This is due to an increase in the probability of mid-banded and three-banded (but not five-banded). For pink snails, an increase in UHI increases the probability of a three-banded shell. The effect of temperature on banding types of pink snails seems to depend on the number of dry days at each location.

**Fig. 8:**
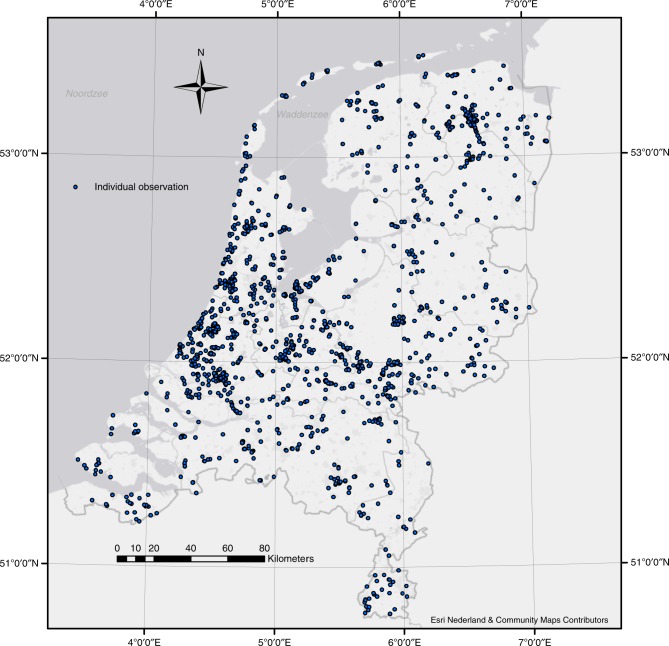
The distribution of the ‘collection' sites. A total of 7977 *Cepaea* spp. were photographed during the SnailSnap-season of 2017. Photographs taken at the same location are shown as a single point

For both yellow, and in particular, pink snails, an increase in the number of dry days in general reduces the probability of a banded versus an unbanded shell. This indicates that, even though the number of dry days is not a significant predictor for the probability of a pink versus a yellow snail (Figs. 5, 8a), the number of dry days does seem to influence pink snails: with increasing numbers of dry days, the proportion of unbanded snails becomes larger (Figs. 7, 8b).

For yellow snails, the proportions of three- and five-banded snails increase with increasing day number. Also for pink snails, the probability of finding a five-banded snail becomes larger later in the season.

**Fig. 9:**
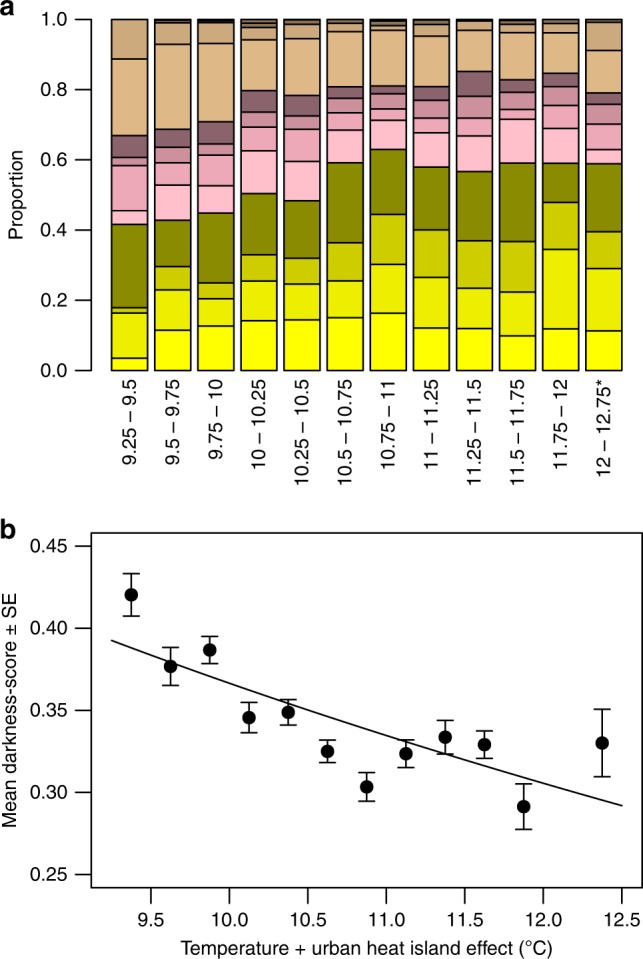
The proportion of colour morphs and average darkness score per temperature + UHI category. **a** Yellow, pink and brown; per colour, from bottom to top: unbanded, midbanded, three-banded, five-banded; *n* = 6809. **b** average darkness-score (*n* = 6838). Each category spans 0.25 °C, starting from 9.25 °C, except the final category, which spans 0.75 °C (three categories were grouped to attain a sample size comparable to the other categories). In **b** the average darkness-score is plotted against the central value of each category. Error bars represent standard errors. There is a negative relationship between the value of temperature+UHI and the average darkness-score (darkness = 1.136–0.334*ln(Temperature+UHI); *F* = 18.09, *p* < 0.01, *R*^2^ = 0.64)

The combined effect of temperature and UHI is shown in Fig. 9. Temperature + UHI explains a high proportion (Fig. 9b, *R*^2^ = 0.64) of the variance in average darkness per temperature + UHI category; on average, darker snails were photographed at colder sites.

### Residual spatial autocorrelation

In models with a spatial aspect, spatial autocorrelation in the residuals could compromise the standard fitting processes^7^. Residual spatial autocorrelation (RSA) might result in underestimated standard errors and, as a consequence, inflated Type I errors^12^. We found that residual spatial autocorrelation was present in the two models we tested (colour all snails (Fig. 5): global Moran’s I = 0.055, *p* < 0.001; banding yellow snails (Fig. 7): global Moran’s I = 0.057, *p* < 0.001). In both models, the distance at which the residuals of two points were no more similar than expected by chance alone was smaller than 500 m.

We ran vector generalized additive models (VGAMs) to account for spatial autocorrelation in these two models. In both cases, adding smoothing splines for location (and day number) reduced RSA. In the VGAM with colour as outcome variable, the global Moran’s I score was reduced to 0.012, yet RSA was still significant (*p* < 0.01). In the VGAM with banding as outcome variable (yellow snails), the global Moran's I score was reduced to 0.009 and was no longer significant.

In both VGAMs, adding the smoothing splines to the model removed the significance of the main effects of temperature and all but one main effect of number of dry days, and most interactions that include these variables. This is not unexpected, as both variables work on a larger geographic scale and are correlated with location (e.g. average temperature decreases with increasing latitude). Significant main effects of UHI, however, remained present. Higher UHI-values were still associated with a higher probability of finding yellow, and a lower probability of finding pink, snails (odds-ratio = 0.720, *z* = −4.0213, *p* < 0.001). Also, higher UHI-values were associated with a higher probability of finding mid- and three-banded yellow snails versus finding an unbanded yellow snail (mid-banded: odds-ratio = 1.495, *z* = 2.8205, *p* < 0.01; three-banded: Odds-ratio = 1.645, *z* = 3.2882, *p* < 0.01). All in all, because temperature and the number of dry days work on a more global scale, and because the effect of UHI remains present in our VGAMs, RSA does not seem to compromise the results of our multinomial regression models (Figs. 2, 4).

## Discussion

We used a citizen-science approach to obtain data on shell colour in Dutch urban and rural *Cepaea nemoralis* populations. As in the earlier, and comparable, Evolution MegaLab^7^, we used data on shell colour to reveal population-genetic patterns that may be related to human-induced environmental change. Our approach, however, differed from the Evolution MegaLab in various ways. First, rather than creating a stand-alone project, we adopted the social and digital network of an existing Dutch citizen science platform, Waarneming.nl (currently around 17,000 active users and 7,000,000 logged observations annually). Second, we minimized the effort required for adding data points: users could use a simple point-and-shoot smartphone app and did not need to supply any additional data. Finally, we specifically aimed at investigating urban evolution, by encouraging participants to record *Cepaea nemoralis* both inside and outside cities. Caution must be observed when using citizen-science-derived data for colour morphs that are sometimes only subtly different, and for which both detectability in the field and interpretation from photographs may be affected by the background colour. However, the two tests we carried out to assess this potential source of error, suggest that these potential biases are not large enough to have affected our overall results and interpretations.

As shell colour and banding pattern in *Cepaea* are almost entirely genetically determined^13^, the differences in urban/non-urban shell coloration among the nearly 10,000 snails that were recorded can be interpreted in the context of urban evolution. We found that shells in urban environments are more likely to be yellow (and less likely to be pink) than in non-urban environments. The logistic regression shows that this is chiefly related to the urban heat island. These results suggest that urban conditions select for yellow snails and against pink snails. The effect is seen in both urban green and urban grey areas, which suggests that the main factor is overall urban air temperature, rather than structural or biological habitat features. There does seem to be a limit to this effect, since at the extreme temperature and UHI end, the proportion ceases to rise.

It is known from experimental work^14–16^ that yellow *Cepaea nemoralis*, either because of a greater albedo (the proportion of received solar radiation that is reflected by an object) or other causes, are better able to survive under high temperature regimes; one study^14^ found that pink-shelled and brown-shelled snails have internal temperatures that are raised compared to yellow. It is therefore plausible that our results reveal natural selection imposed by the urban heat island on the (recessive) yellow allele for the shell ground colour gene in *C. nemoralis*.

The results for shell banding are somewhat more complex. Dark spiral bands on the shell reduce a snail’s albedo and are expected to raise the snail’s body temperature^14^, and would, therefore, be expected to be selected against in the urban heat island. However, we found that this is only true for 5-banded morphs. The other main banding morphs (mid-banded and three-banded) in fact increase under urban conditions as well as under increasing temperature. Longitudinal studies of the evolutionary response of *C. nemoralis* to climate warming obtained similar results^7,17^. There are several possible explanations for this counter-intuitive effect. First of all, it is known that shell banding in *C. nemoralis* interacts with environmental factors such as shell-crushing predators: banded snails are stronger^18^ and, in certain habitats, better camouflaged^19,20^. Although we could not test for them, these evolutionary pressures may also differ in urban versus non-urban contexts^21^. Furthermore, it is not impossible that the bands affect thermoregulation in yet underexplored ways. In the related *Theba pisana* it was found^22^ that dark bands on a light-coloured shell enhance cooling after an exposure to heat, possibly by creating differential airflow between dark and light areas, and by more rapid heat dissipation from the stripes. If such effects exist in *C. nemoralis* as well, they could combine with the greater albedo in yellow shells to select for yellow urban snails that have the exposed, top part of the shell unbanded (and therefore more reflective), while bearing bands on the underside (i.e., yellow mid- and three-banded) that may enhance cooling.

Although our results are most consistent with a response to temperature, we cannot fully rule out an additional effect of camouflage. Even in urban green spaces, overall backgrounds may be different in colour and structure than in rural green spaces. We are currently embarking on experimental studies to disentangle the agents of selection on shell colour in urban environments.

In summary, we show that a simple smartphone app, linked to a citizen science web platform, enables effective monitoring of phenotypic change in the urban heat island, probably as a result of natural selection. Our results concern the data collected in the first year, but the app continues to generate data, which could be used to confirm our results in future years.

The system we describe could also be expanded to other organisms that are known to display easily observable phenotypic evolution in urban contexts, such as plumage coloration in rock pigeons, *Columba livia*^23,24^, and male “necktie” width in the great tit, *Parus major*^25^. This would allow the development of a continuous monitoring system of urban evolution in multiple organisms — especially if current attempts to replace human validation with artificial intelligence in citizen science apps^26^ are successful.

## Methods

### SnailSnap

A smartphone application (SnailSnap) was developed for Android-devices. The app is available for download, free of charge, at Google Play Store (https://play.google.com/store/apps/details?id=nl.zostera.slakkenschieter). It was designed as a convenient and accessible method to upload images of live, adult *Cepaea nemoralis* to Waarneming.nl (the Dutch version of Observation.org). The SnailSnap project officially began on April 1st, 2017. Prior to this date, we sent out a press release. We also developed a website (www.snailsnap.nl), gave interviews on national radio, and handed out flyers at nature-related events. During the entire course of the project, we used the public social media channels (Facebook and Twitter) of the Netherlands natural history museum Naturalis, and the online newsletters of Waarneming.nl and insect knowledge centre EIS to inform the general public.

Participants were instructed (via the website and instructions incorporated into the app) on how to find, recognize, and photograph adult *C. nemoralis*. They were asked to take a single photograph per snail, to include only one snail per picture, and to take the pictures at such an angle that the lip and all bands on the shell were visible. Images and the associated GPS-coordinates were automatically uploaded to the Waarneming.nl server as soon as the participant's smartphone connected to a wifi-network. Alternatively, app-users could send the pictures to the server via mobile internet using the Send-button that was incorporated in the app.

Participants could also upload pictures to the server via the Waarneming.nl website and its apps iObs and Obsmapp. All pictures that were uploaded were then handled by a group of ten validators, who checked the correct identification as *C. nemoralis*, and attributed the correct colour and banding code to each snail. All snails with a visible dark lip were included in the data set. *Cepaea hortensis*, which can be distinguished from *C. nemoralis* by its white lip, is rare in most parts of the Netherlands. Therefore, only those pictures without a visible dark lip were removed from the data set if they were taken in one of three known areas where *C. hortensis* is relatively common (namely, the south of the province of Limburg [latitude < 51.2], the northeast of the province of Groningen [latitude > 53, longitude > 6.5] and the area east and north-east of Nijmegen [51.8 < latitude < 52, longitude > 5.75]).

We chose October 15^th^, 2017, as end date (in the Netherlands, *C. nemoralis* starts hibernating around this date). Not all uploaded images could be classified to colour morph. Some were very unclear or had been taken from an inappropriate angle. Sometimes single snails were photographed more than once (in this case, only one of the pictures was classified). Quite a large number of pictures had been taken from an apical angle, hence the lower bands (4^th^ and 5^th^) were not visible. Snails on such pictures were classified into a special group of codes and used only for analyses of shell ground colour (not banding). The remaining observations were relatively evenly distributed over the country (Fig. 8).

### Accuracy of the SnailSnap data

To test the accuracy of the colour and banding classification by validators, we double-checked 240 snails (60 snails starting at four random dates, i.e., 1^st^ of August, 15^th^ of July, 18^th^ of September, and 28^th^ of April). We found that the banding pattern was only misclassified once. In 33 out of the 240 cases, however, our colour classification did not match the classification by the validator. The most common discrepancies were dark pink snails classified as brown, and multi-banded pink snails as yellow. All validators had been given instructions on how to classify snails. Also, during the course of the project, validators who occasionally misclassified snail colours had been asked to revisit the instruction documents and/or to adjust the settings of their computer screen. Furthermore, in several cases probably we were the ones to misclassify a snail. Mainly due to the variation within each of the three main colour categories, assigning a colour to a snail is not always straightforward. This is especially true for people who are not experienced with studying *Cepaea*. Although it is clear that the current colour classification method is not perfect, it is probably more accurate than the method used in the Evolution Megalab, a previous citizen science project on *Cepaea*^7^.

Because citizen scientists did not use standardized search techniques, in certain habitats/backgrounds, some colour morphs might have been easier to locate than others. Therefore, certain colour morphs might have been systematically overlooked in specific habitats. In addition, categorization into colour morphs from pictures might be influenced by light conditions and visual background, which, in turn, is likely related to the habitat in which the snails were found. To investigate the extent to which these issues might influence our results, we performed two small experiments. In both experiments, we used empty *C. nemoralis* shells marked at the bottom with a unique number.

In experiment 1, we selected 42 shells. Proportions of colour morphs roughly corresponded to the proportions found in the Netherlands (Fig. 2b). Four volunteers were grouped into two pairs. Each pair visited three locations in Leiden (see Fig. 2a). At the first location, the shells were shown to the volunteers. One of the volunteers was asked to distribute the shells, apex up, within a marked area of five by five metres. The volunteer was instructed to first pick a spot to put a shell, and then blindly select a random shell from a non-transparent bag. The other volunteer was subsequently asked to search for the shells. Every time the volunteer found a shell, he or she mentioned the number written on the shell and put the shell into a bag. It was recorded after how much time each shell was found. Search time was limited to ten minutes per round. After the first round at each location, the volunteers switched roles. Each volunteer thus once distributed shells and once searched shells at each location. In experiment 2, 84 shells were selected, making sure the most common colour morphs were included (Supplementary Table 1). On one day (weather: overcast with some rain), these 84 shells were each photographed three times, against soil/vegetation, tree, and concrete backgrounds. Pictures were taken using a Samsung Galaxy J6 (no filter) and were not edited. All pictures were sent digitally to M.S., who assigned a colour (yellow, pink of brown) to the shell on each photograph. After categorizing all pictures, M.S. also assigned colours to the actual shell specimens. In addition, N.A.G.K. also categorized the actual shell specimens.

During the course of the project, we noticed that consecutive pictures taken in the app by the same user sometimes had the same GPS-coordinates. It transpired that certain conditions (a low quality mobile phone, cloudy weather, tall buildings) could cause a GPS-fix to fail. Pictures would then be associated with the last successful GPS-fix until a new GPS-fix was made. Although this problem is, to some extent, unavoidable (highly accurate GPS-fixes could take unacceptably long under some conditions), the app was updated to improve this condition on September 11. We have no way of knowing to what extent this GPS-problem has influenced the accuracy of locality data for the observations, but we expect it to be negligible.

### Data analysis

All data on shell colour, banding, and location were exported from the Waarneming.nl server to a spreadsheet. The 7,868 individual data points, each representing a single snail, were then imported into an ArcGIS v10.2.2 (Environmental Systems Research Institute, Redlands, CA) project. Using the tool “Extract Multi Values to Points” and the “Join data” dialogue box (join by location), environmental and climatic geographical data were added to the data points based on the location of each point. We used the following geographical data. (1) ‘Urban Heat Island effect’, average difference in air temperature between urban and surrounding rural areas in summer; based on relationships between UHI and the combination of wind speed, population density, the presence of open water, the presence of vegetation and the percentage of soil sealing (°C, grid, 10 × 10 metres; source: Rijksinstituut voor Volksgezondheid en Milieu (RIVM), https://data.overheid.nl/dataset/49121-stedelijk-hitte-eiland-effect–uhi–in-nederland, downloaded 21^st^ December, 2017); (2) ‘Temperature’, average yearly temperature in the period 1981–2010, based on data from 28 weather stations (°C, grid, 1000 × 1000 m; source: Koninklijk Nederlands Meteorologisch Instituut (KNMI)

https://data.knmi.nl/datasets, downloaded: 21st December 2017); (3) ‘Number of dry days’, Average number of dry days per year in the period 1981–2010, based on approximately 300 observations done by volunteers (grid, 1000 × 1000 m; source: KNMI https://data.knmi.nl/datasets, downloaded: 21^st^ December 2017); (4) ‘Land use’, land use data of the Netherlands, based on a digital topographic map (Top10NL, 1:10.000) and aerial photographs, data collected during the summer of 2012 (land use, vector; source: ZBO, Centraal Bureau voor de Statistiek, https://data.overheid.nl/dataset/58878-bestand-bodemgebruik-2012, downloaded: 21^st^ December 2017).

All statistical analyses were performed in R (version 3.4.3 [ref. ^27^.]). To compare proportions of colour morphs from different habitats, we included only snails with a complete banding code that fit into one of the four main banding types (unbanded, midbanded, three-banded, and five-banded; 6,809 in total). Based on the GPS-coordinates, snails were assigned to one of four habitat types: (1) agricultural land (*n* = 832), (2) nature (including forests; *n* = 888), (3) urban green areas (urban parks and forests, sport and recreational areas; *n* = 817) and (4) urban “grey” areas (residential, commercial, and industrial areas; *n* = 4008). The proportions of snails of different colours and/or banding types were compared between habitat types using Chi-squared tests.

Multinomial logistic regression models with shell colour and banding type as outcome variable were run using the “multinom” function from the nett package^28^. Odds-ratios, *z*-values and *p*-values were obtained via the “tidy” function from the broom package^29^. All predictor variables were centred around zero (centred X = X – mean(X); centred predictor values were calculated for each model separately). To interpret marginal effects and significant interaction terms, effects were displayed using the effects package^30^.

To visualize the combined effect of temperature and UHI (temperature + UHI), this variable was divided into categories and proportions of colour morphs were calculated per category.

To study the combined effect of shell colour and banding patterns, for each snail with a complete banding code (*n* = 6,838), a score was calculated based on the known thermal properties of colour morphs^14^. Using yellow unbanded snails as the baseline, the expected internal temperature increases of the other morphs were calculated by adding 0.3 °C for pink, 0.6 °C for brown, 0.07 °C for each band and 0.03 °C for each band fusion. The resultant expected temperature increase was used as “darkness score”^31^. For temperature + UHI, average darkness scores were calculated per category. These average darkness scores were plotted against the central variable value of each category and a regression line was fitted.

To illustrate the interaction between temperature and UHI, colour morph proportions were calculated per UHI-category for snails twice: once for sites with an average temperature below or equal to 10 °C (the median temperature) and once for sites with an average temperate above 10 °C.

To assess the presence of residual spatial autocorrelation (RSA), we calculated global and local Moran’s I coefficients for the residuals of the reference groups of two of our models, namely: colour all snails (Fig. 5), reference: yellow; and banding yellow snails (Fig. 7), reference: unbanded. Distance matrices were created using the “distm” function from the geosphere package (Haversine great-circle distance^32^). Using inverse distance matrices, global Moran’s I scores were calculated with the “Moran.I” function from the ape package^33^. Local Moran's I scores and correlograms were calculated using the “correlog” function from the ncf package^34^.

To account for spatial autocorrelation in these two models, we ran vector generalized additive models (VGAMs, family = multinomial) including all environmental/climatic predictor variables (UHI, temperature and number of dry days, and all possible interactions) plus spline smooth terms (O’Sullivan splines) for latitude, longitude, the interaction between latitude and longitude, and the number of days since the start of the project. These models were run using the “vgam” function, and marginal effects were calculated using the “margeff” function (both from the VGAM package^35^).

### Reporting summary

Further information on research design is available in the Nature Research Reporting Summary linked to this article.

## Supplementary information

Supplementary items

Reporting Summary

## Data Availability

Raw data are available from Dryad^36^ at doi:10.5061/dryad.7rh631v. The dataset includes primary data and metadata on citizen-science derived shell colour for *Cepaea nemoralis*, in relation to adaptation to the urban heat island. Each row is a single snail, photographed in the field by a participant.
