# Supplementary material for: Snail shell colour evolution in urban heat islands detected via citizen science
Source: Commun Biol. 2019 Jul 19;2:264. doi: 10.1038/s42003-019-0511-6 (PMC6642149; doi:10.1038/s42003-019-0511-6)
Supplement: Supplementary file 1 — Supplementary items [file 42003_2019_511_MOESM1_ESM.pdf]

Supplementary table 1. The 84 shells used in experiment 2, as classified by MS based on the actual shell specimens.

**Colour**	**Banding**	**Number of shells used**
Yellow	unbanded	13
	midbanded	11
	three-banded	10
	five-banded	10
Pink	unbanded	13
	midbanded	6
	three-banded	8
	five-banded	5
Brown	Unbanded	8

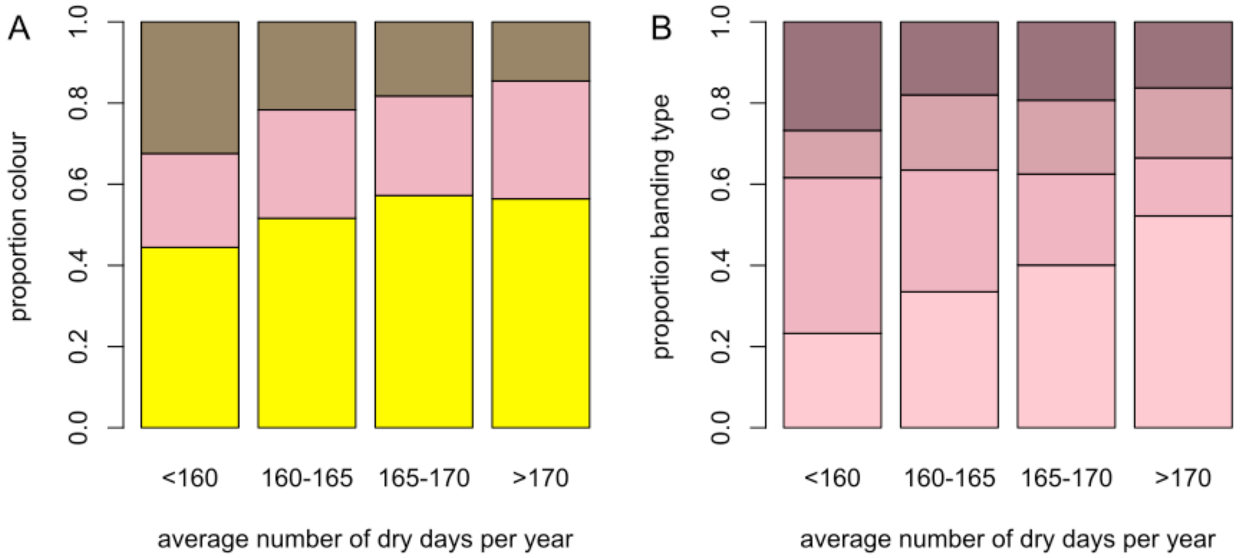

Supplementary figure 1. Proportion of snails of different colours (A; yellow, pink, and brown) and banding types of pink snails (B; from bottom to top: unbanded, mid-banded, three-banded, and five-banded) per category of the number of dry days. The average number of dry days per year was divided into four categories based on the sample distribution (sample size per category: 916, 2,547, 2,722, and 1,683 for A; 172, 567, 544, and 412 for B). In line with the results from the multinomial regression models, the proportion of brown snails decreases (A), and the proportion of pink unbanded snails increases (B), with an increasing number of dry days.
