# Supplementary material for: Snail shell colour evolution in urban heat islands detected via citizen science
Source: Commun Biol. 2019 Jul 19;2:264. doi: 10.1038/s42003-019-0511-6 (PMC6642149; doi:10.1038/s42003-019-0511-6)
Supplement: Supplementary file 2 — Reporting Summary [file 42003_2019_511_MOESM2_ESM.pdf]

nature research

Corresponding author(s): Menno Schilthuizen, June 2nd, 2019

Reporting Summary

Nature Research wishes to improve the reproducibility of the work that we publish. This form provides structure for consistency and transparency in reporting. For further information on Nature Research policies, see Authors & Referees and the Editorial Policy Checklist.

Statistical parameters

When statistical analyses are reported, confirm that the following items are present in the relevant location (e.g. figure legend, table legend, main text, or Methods section).

n/a	Confirmed	
☐	☒	The exact sample size (*n*) for each experimental group/condition, given as a discrete number and unit of measurement
☐	☒	An indication of whether measurements were taken from distinct samples or whether the same sample was measured repeatedly
☐	☒	The statistical test(s) used AND whether they are one- or two-sided<br>*Only common tests should be described solely by name; describe more complex techniques in the Methods section.*
☐	☒	A description of all covariates tested
☐	☒	A description of any assumptions or corrections, such as tests of normality and adjustment for multiple comparisons
☒	☐	A full description of the statistics including central tendency (e.g. means) or other basic estimates (e.g. regression coefficient) AND variation (e.g. standard deviation) or associated estimates of uncertainty (e.g. confidence intervals)
☐	☒	For null hypothesis testing, the test statistic (e.g. *F*, *t*, *r*) with confidence intervals, effect sizes, degrees of freedom and *P* value noted<br>*Give P values as exact values whenever suitable.*
☒	☐	For Bayesian analysis, information on the choice of priors and Markov chain Monte Carlo settings
☒	☐	For hierarchical and complex designs, identification of the appropriate level for tests and full reporting of outcomes
☒	☐	Estimates of effect sizes (e.g. Cohen's *d*, Pearson's *r*), indicating how they were calculated
☐	☒	Clearly defined error bars<br>*State explicitly what error bars represent (e.g. SD, SE, CI)*

Our web collection on statistics for biologists may be useful.

Software and code

Policy information about availability of computer code

Data collection	Observations were collected using the SnailSnap-app, the online interface of Waarneming.nl and the apps iObs and ObsMapp. Colour morph codes were attributed online using the Waarneming.nl interface. All observations were exported from the Waarneming.nl server to a spreadsheet.
Data analysis	GIS-analyses were performed using ArcGIS v10.2.2 (Environmental Systems Research Institute, Redlands, CA). Using the tool "Extract Multi Values to Points" and the "Join data" dialog box (join by location), environmental and climatic geographical data were added to the data points based on the location of each point. All statistical analyses were performed in R (version 3.4.3). Multinomial logistic regression models were run using the "multinom" function from the nett package. Odds-ratios, z-values and p-values were obtained via the "tidy" function from the broom package. To interpret marginal effects and significant interaction terms, effects were displayed using the effects package.

For manuscripts utilizing custom algorithms or software that are central to the research but not yet described in published literature, software must be made available to editors/reviewers upon request. We strongly encourage code deposition in a community repository (e.g. GitHub). See the Nature Research guidelines for submitting code & software for further information.

Data

Policy information about availability of data

All manuscripts must include a data availability statement. This statement should provide the following information, where applicable:

- Accession codes, unique identifiers, or web links for publicly available datasets
- A list of figures that have associated raw data
- A description of any restrictions on data availability

The raw data are available from the public citizen science platform waarneming.nl. A tabel with the data we extracted, and to which land-use and other parameter values have been added, will be deposited in Dryad.

Field-specific reporting

Please select the best fit for your research. If you are not sure, read the appropriate sections before making your selection.

☐ Life sciences ☐ Behavioural & social sciences ☒ Ecological, evolutionary & environmental sciences

For a reference copy of the document with all sections, see nature.com/authors/policies/ReportingSummary-flat.pdf

Ecological, evolutionary & environmental sciences study design

All studies must disclose on these points even when the disclosure is negative.

Study description	We used a citizen-science approach to study the effects of urban heat on genetically-determined shell colour in the land snail Cepaea nemoralis in the Netherlands. We used smartphone applications to obtain colour data on almost 8,000 snails throughout the country. Using multinomial regression, we tested whether temperature, the Urban Heat Island effect and precipitation (the number of dry days), and interactions between these factors, influence the probability of finding certain shell colour morphs at specific locations. Furthermore, we used Chi-squared tests to compare proportions of colour morphs between habitat types.
Research sample	Citizen scientists were asked to find and photograph live, adult Cepaea nemoralis in the Netherlands, from all possible habitats.
Sampling strategy	Citizen scientists were asked to take as many pictures of individual snails as they could find, both within and outside urban areas. We used (social) media to inform the general public and to encourage people to participate.
Data collection	Data were collected by citizen scientists using mobile apps.
Timing and spatial scale	The citizen science project started on April 1st 2017 and ended on October 15th 2017, which roughly corresponds to the entire Cepaea nemoralis season in the Netherlands.
Data exclusions	Not all uploaded images could be classified to colour morph. Some were very unclear or had been taken from an inappropriate angle. Sometimes single snails were photographed more than once. In this case, only one of the pictures was classified. Those pictures without a visible dark lip (animals on these pictures could belong to a related species, Cepaea hortensis) were removed from the data set if they were taken in one of three known areas where C. hortensis is relatively common (namely, the south of the province of Limburg [latitude < 51.2], the northeast of the province of Groningen [latitude > 53, longitude > 6.5] and the area east and north-east of Nijmegen [51.8 < latitude < 52, longitude > 5.75]).
Reproducibility	The smartphone-based collecting of data is ongoing. Our results concern only the first season (2017). The experiment can be repeated by carrying out a similar procedure on data accumulated in subsequent seasons (2018 and onwards).
Randomization	Citizen scientists were encouraged to take a picture whenever they would find or encounter a snail. They were instructed on how to recognize Cepaea nemoralis. It was made clear, via text and images, that Cepaea nemoralis shells are highly polymorphic and can be yellow, pink or brown and banded or unbanded.
Blinding	Data were collected by citizen scientists. Almost all colour morph classifications were done by a group of volunteers (Niels Kerstes and Menno Schilthuizen also contributed to the classification of the snails).

Did the study involve field work? ☒ Yes ☐ No

Field work, collection and transport

Field conditions	Diverse
Location	The entire country of the Netherlands
Access and import/export	No samples were imported or exported.
Disturbance	Disturbance was minimized by taking pictures of the snails instead of collecting them.

Reporting for specific materials, systems and methods

Materials & experimental systems

n/a	Involved in the study	
☒	☐	Unique biological materials
☒	☐	Antibodies
☒	☐	Eukaryotic cell lines
☒	☐	Palaeontology
☐	☒	Animals and other organisms
☒	☐	Human research participants

Methods

n/a	Involved in the study	
☒	☐	ChIP-seq
☒	☐	Flow cytometry
☒	☐	MRI-based neuroimaging

Animals and other organisms

Policy information about studies involving animals; ARRIVE guidelines recommended for reporting animal research

Laboratory animals	The study did not involve laboratory animals
Wild animals	Citizen scientists were asked to take pictures of live, adult Cepaea nemoralis individuals. No animals were collected and/or used in experiments.
Field-collected samples	The study did not involve field-collected samples
